# Object correspondence in audition echoes vision: Not only spatiotemporal but also feature information influences auditory apparent motion

**DOI:** 10.3758/s13414-025-03175-7

**Published:** 2025-12-04

**Authors:** Meike C. Kriegeskorte, Bettina Rolke, Elisabeth Hein

**Affiliations:** Department of Psychology, University of Tübingen, Schleichstrasse 4, D – 72076, Tübingen, Germany

**Keywords:** Perceptual organization, Grouping, Object correspondence, Apparent motion, Ternus display, Auditory perception

## Abstract

A crucial ability of our cognition is the perception of objects and their motions. We can perceive objects as moving by connecting them across space and time. This is possible even when the objects are not present continuously, as in the case of apparent motion displays like the Ternus display, consisting of two sets of stimuli, shifted to the left or right, separated by a variable inter-stimulus interval (ISI). This is an ambiguous display, which can be perceived as both stimuli moving uniformly to the right (group motion) or one stimulus moving across the stationary center stimulus (element motion), depending on which stimuli are connected over time. Which percept is seen can be influenced by the ISI and the stimulus features. Previous experiments have shown that the Ternus effect also exists in the auditory modality and that the auditory Ternus is also dependent on the ISI. This is a first indication that correspondence might work similarly in the visual and auditory modality. To test this idea further, we investigated whether the auditory Ternus effect is dependent on the stimulus features by creating a frequency-based bias using a high and a low sinewave tone as Ternus stimuli. This bias was compatible either with the element-motion or with the group-motion percept. Our results showed an influence of this feature bias in addition to an ISI effect, suggesting that the visual and the auditory modalities might both use the same mechanism to connect objects across space and time.

## Introduction

Organizing and interpreting acoustic information is a central aspect of auditory perception. The brain can break down complex acoustic environments into different sound sources and identify meaningful events (e.g., Bregman, 1990). For this purpose, the acoustic stimuli must be organized in auditory streams to clearly filter out individual sound sources and block out other irrelevant noises. This ability is known as *auditory scene analysis* (Bregman, 1990) and is used, for example, in the *cocktail party effect* (Cherry, 1953): One’s own name is so relevant that it is selected and perceived despite many other conversations that are simultaneously arriving at the ear. This ability of auditory selection is crucial for understanding and interpreting the auditory environment. Research in this area has shown that auditory selection can be influenced by various physical characteristics of the sound, such as pitch, loudness and temporal structure (e.g., Bregman, 1990; Bregman & Campbell, 1971; Cusack & Carlyon, 2004; Darwin & Carlyon, 1995; Griffiths & Warren, 2004; Shamma et al., 2011; van Noorden, 1975). Understanding auditory selection is essential for investigating how the brain processes and integrates complex sensory information.

Individual acoustic elements can perceptually merge to form a larger unit that can be perceived as an auditory object (e.g., Griffiths & Warren, 2004). Research often focuses on stationary objects, but objects in the environment are often only stationary if we consider a fixed point in time. But when observing an object (or a person) over time, it usually shifts in space, because the object or the observer is moving. We perceive continuous motion, despite the sampling of the sensory input not being continuous and the motion path of an object not being always fully visible, for example if an object moves behind other objects. Furthermore, we can even perceive motion between objects that do not really move: if two objects are presented at two different locations one after the other, we perceive apparent motion between them, as long as the spatial and temporal distance is suitable, i.e., not too long/far or too short/close (e.g., Korte, 1915; Wertheimer, 1912). (Apparent) motion is perceived, because the viewer establishes correspondence between two stimuli that appear at different times in different locations (e.g., Ullman, 1979). Ascertaining which objects are linked to each other and establishing correspondence between the right instances of an object is referred to as the correspondence problem (Ullman, 1979). Only if we establish correspondence between the right objects, can we create coherent representations of objects in motion. Apparent motion can not only be perceived for visual objects, but also for sounds (e.g., Lakatos, 1993). Lakatos (1993), for example, showed that people can perceive motion between the sound of loudspeakers at different locations that is dependent on the spatial and temporal distance between the sounds. Thus, the correspondence problem also exists for auditory apparent motion, where correspondence has to be established between the right sound sources, in order to perceive objects and their sounds in a coherent way.

Although it has been shown that apparent motion is present in the auditory as well as the visual modality, little is known about how correspondence is established, i.e., how the correspondence problem is solved in the auditory modality. Many studies concerning the correspondence problem have been done in the visual modality by using different ambiguous apparent motion displays, as for example the Ternus display (Ternus, 1926). In that display, two stimuli, in vision usually circles, are presented to the left and at the center of a computer monitor (frame 1, a frame contains everything that is presented on the monitor and stays the same for a specific amount of time), followed by a variable pause (inter-stimulus interval, ISI) and then one stimulus is presented at the same center position as in the frame before and another one to the right of that center stimulus (frame 2). This display can be perceived either as group motion, when both stimuli appear to move one position to the right, or as element motion, when the outer stimulus appears to jump over the center stimulus to the right. Which motion is perceived depends on the correspondence established between the stimuli.

Using the Ternus display, researchers have examined whether spatiotemporal and feature information influence how the correspondence problem is solved. One major result is that with a short ISI between the two frames element motion is predominantly perceived and with a long ISI group motion is predominantly perceived (e.g., Pantle & Picciano, 1976; Petersik & Pantle, 1979). This showed that spatiotemporal factors can affect the perceived correspondence between the stimuli. Based on these findings, motion-based theories have been developed to explain the correspondence mechanism, such as a low-level mechanism that determines motion energy based on the output of simple motion detectors or spatiotemporal filters (e.g., Adelson & Bergen, 1985; van Santen & Sperling, 1985). According to this view, spatiotemporal information is a dominant factor for solving correspondence. If feature information contradicts spatiotemporal information, the perceptual system will accommodate this difference (e.g., Burt & Sperling, 1981; Kolers & von Grünau, 1976), for example, by perceiving an object as changing color or shape while moving.

In addition to spatiotemporal influences, the features of the visual stimuli (e.g., color, shape, and luminance) also influence the perceived motion (e.g., Hein & Moore, 2012; Kramer & Yantis, 1997; Petersik & Rice, 2008). Hein and Moore (2012), for example, created two different types of bias, a group bias, in which the first stimulus of each frame was presented with the same feature, for example, red color, and the second stimulus with a different feature, for example, blue color. They compared this condition with an element bias, in which the features of the outer stimuli in each frame were different from the center stimuli. If the feature information influences correspondence, then stimuli with the same features should more likely be connected and as a result observers should perceive more group motion with the group bias and more element motion with the element bias. That is exactly what Hein and Moore (2012) found (see also Kramer & Yantis, 1997; Petersik & Rice, 2008). In addition, they found that the group bias was less dependent on the ISI than the element bias, suggesting that the group bias can create a stronger feature bias than the element bias. To explain the influence of feature information on correspondence, object-based theories have been developed that suggest, in contrast to motion-based theories, that the feature information of the objects, such as their color or shape, but also perceived features, such as lightness or perceived size, is at least as important as spatiotemporal information. Following these theories, correspondence is resolved on the basis of all available information about an object, including the perceptual organization or the relations between the different elements of the display, and motion is perceived as a consequence of which objects are most similar to each other (e.g., He & Ooi, 1999; Hein & Cavanagh, 2012; Hein & Moore, 2012, 2014; Kramer & Rudd, 1999; Ramachandran et al., 1998).

**Fig. 1:**
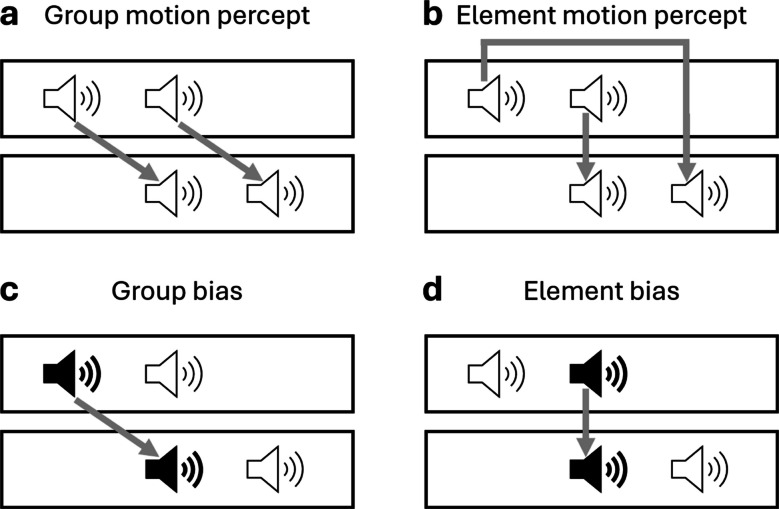
Classic and biased auditory Ternus display. The auditory Ternus display allows for an ambiguous apparent motion percept. We created a feature bias with two different tone frequencies (here illustrated with black and white loudspeakers). (**a**) Illustration of the group-motion percept, when both stimuli appear to move uniformly to the right (or left). (**b**) Illustration of the element-motion percept, when the outer stimulus seems to jump over the middle stimulus to the right (or left) side. The middle stimulus is perceived as continuous and stationary. (**c**) Example of a group feature bias: the frequency of the first tone of each frame is different from the frequency of the second tone, the frequencies being compatible with the group-motion percept. (**d**) Example of an element feature bias: the frequency of the outer tones is different from the center tones, compatible with the element-motion percept

While most research on the Ternus display has been done in the visual modality, more recently it has been shown that the Ternus effect also exists in other modalities. Wang et al. (2014) established an auditory version of the Ternus display in which sounds are used instead of circles. They presented four tones through three speakers that were placed horizontally on a desk: the first two tones were presented to the left and at the center, followed by the ISI, after which the second two tones were presented at the center and to the right (see Fig. 1). We call the tone sequence before and after the ISI a frame as in the visual modality, although there is no presentation on a screen. In this auditory Ternus display, two different sound percepts are possible, either two sounds moving together from one (loudspeaker) location to the other or one sound that appears to move across the center sound that is perceived as stationary (Wang et al., 2014). As in the visual modality, the authors found that in the auditory modality the temporal length of the ISI influenced whether group or element motion was perceived, in particular, with the longer ISI more group motion was perceived. Harrar and Harris (2007) showed that the Ternus display also works in the tactile modality. They created a tactile version of the Ternus display, using pins under the index, middle, and ring fingers. The pins were pushed out to present a stimulus. The first two pushes were presented at the index and middle fingers, followed by the ISI, and then the second two pushes were presented at the middle and ring fingers. In line with the results in the visual and auditory modalities, the authors found the same spatiotemporal influence, i.e., the longer the ISI, the more group motion was perceived. Thus, in all three modalities, vision, audition, and touch, the apparent motion percept in the Ternus display is influenced by the ISI, suggesting that correspondence can be based on spatiotemporal information in all these different modalities.

The finding that spatiotemporal factors influence the apparent motion percept in a very similar way in different modalities might suggest that similar (or even the same) mechanisms are used to connect objects across space and time within these modalities. This assumption, however, is based on the spatiotemporal relations (i.e., the ISI) alone. A stronger statement regarding the mechanisms contributing to solving the correspondence problem in different modalities could be obtained by analyzing further factors which have been shown to influence the visual modality. As described above, correspondence in the visual modality is strongly influenced by feature similarity (e.g., Hein & Moore, 2012; Kramer & Yantis, 1997). The present study aimed to investigate whether feature similarity is used to resolve correspondence for auditory apparent motion. To the best of our knowledge this question has never been investigated before. We used an auditory Ternus display (see Fig. 1a and b) and in Experiment 1 we first tried to replicate the ISI effect found by Wang et al. (2014). In Experiments 2 and 3 in addition to the ISI, we manipulated the frequency of the tones to create two types of feature bias (Fig. 1c and d): Similar to Hein and Moore (2012) in the visual modality, we created a group bias, in which the frequency of the first tone of each frame differed from the frequency of the second tone. We also created an element bias, in which the frequency of the outer tones differed from the center tones. Besides these two biases, there was a no-bias condition in which all tones had the same frequency. This feature bias was presented in a mixed design in which all trials occurred in a random sequence in Experiment 2. In Experiment 3 we tested if the effect of the feature bias is robust across different presentation modes by using a blocked design, in which the feature bias remained the same within a block of trials. Based on the results of Wang et al. (2014), we expected that observers would perceive more group motion the longer the ISI was. In addition, if correspondence is established between stimuli whose features are the same, the group bias should lead to more group-motion percepts and the element bias should lead to more element-motion percepts, as is found in the visual modality (e.g., Hein & Moore, 2012; Kramer & Yantis, 1997; Petersik & Rice, 2008). Our results will provide new insights into how the brain establishes correspondence between auditory objects and thus how it perceives auditory objects as moving over time.

## Experiment 1: Spatiotemporal factor

In this experiment, we investigated how spatiotemporal factors influence the correspondence process in an auditory Ternus display, trying to replicate the ISI effect found by Wang et al. (2014). We tested six ISIs ranging from 0 to 300 ms. For the auditory Ternus to work, the second stimulus in each frame must be a little bit delayed (within-frame interval, WFI), as otherwise the two stimuli are merged into one stimulus percept (*precedence effect*; Litovsky et al., 1999). It is important to avoid the merging of the sounds, as the perception of the Ternus motion is only possible if two separate auditory objects are perceived in each frame. If due to merging only one sound is perceived in each frame, no Ternus motion, only simple apparent motion between two auditory objects, can be perceived across frames. To assess whether participants perceived Ternus motion or not, in addition to element and group motion, we introduced the response category “no (Ternus) motion.” We manipulated the WFI in three steps (10, 80, and 100 ms). As the 10-ms WFI should be too short to avoid sound mixing, while the 80- and 100-ms WFI should be sufficient, we expected more no-motion percepts in the 10-ms WFI condition than in the other WFI conditions. Most importantly, based on Wang et al. (2014), we expected to find more group-motion and less element-motion percepts with increasing ISI.

### Materials and methods

#### Participants

The sample size in Experiment 1 was based on Wang et al. (2014), who used 14 (Exp. 1a), 12 (Exp. 1b), and 13 (Exp. 2) participants in their study. To ensure that the sample size of Wang et al. (2014) was sufficient, we additionally performed a power analysis using RStudio, converting the F-values and degrees of freedom from the study by Wang et al. (2014) for the main effect of ISI. We obtained a sample size of two as being necessary to achieve .8 power, assuming an alpha of .05, showing that the study from Wang et al. (2014) was not underpowered. Participants who showed an inversive function of ISI, i.e., the atypical pattern of more element motion for longer ISIs, were replaced to allow for a better comparison with visual studies, which use the same exclusion criterion (e.g., Hein & Moore, 2012; Kramer & Yantis, 1997). Based on that exclusion criterion, three participants were replaced in Experiment 1. The final sample consisted of 14 participants (ten females; aged between 19 and 32 years, average age 24.29 years; 13 right-handers). All participants received either course credit or money as compensation for their participation. The participants reported normal or corrected-to-normal visual and auditory acuity. None of the participants took part in more than one of the experiments. The ethics committee of the University of Tübingen approved the experiments in this study (reference number: Labor_Rolke_2022_0413_252), and all of the participants signed an informed consent form in accordance with the ethical guidelines of the Declaration of Helsinki (World Medical Association, 2013).

#### Equipment

**Fig. 2:**
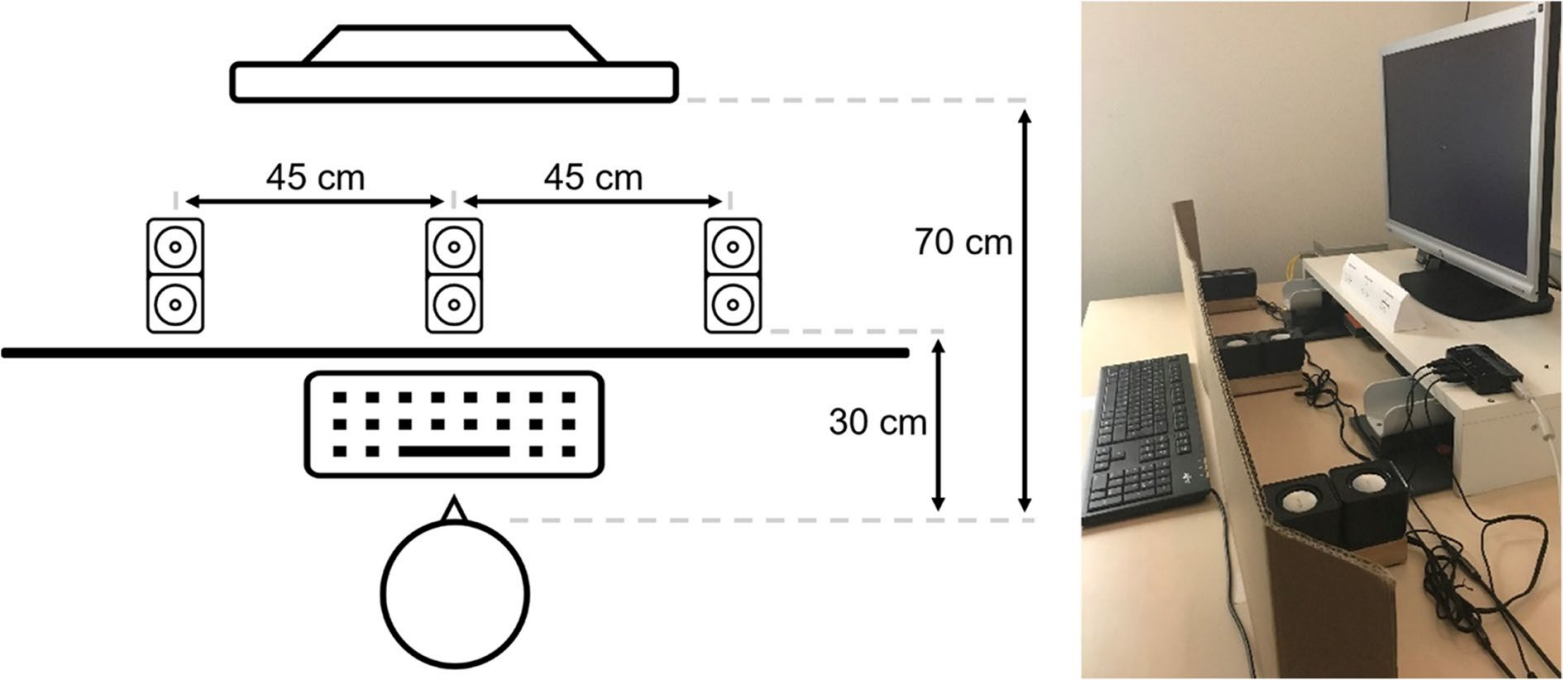
Set-up in all three experiments. There were three loudspeaker sets, each with two loudspeakers, in front of the participants: Left, center, and right. The speakers were each 45 cm apart and 30 cm away from the participants. Between the keyboard and the loudspeakers was a cardboard sign to block the view. The viewing distance to the computer screen was 70 cm

Three mini stereo loudspeaker sets (Trust Leto Compact 2.0 Speaker Set; audio connection: 3.5 mm; height: 73 mm; width: 62 mm; depth: 55 mm) with two loudspeakers per set were placed on a horizontal line in front of the participants (distance: 30 cm). They were all facing upwards (see Fig. 2). One set of speakers was positioned on the left, one directly at the center, and one on the right (distance between the sets: 45 cm). The speakers within a set were positioned directly behind each other. The speakers were presented behind a cardboard sign that blocked the view of the loudspeakers and was intended to prevent a location-dependent expectation. Behind the loudspeakers was a computer screen (viewing distance: 70 cm; resolution: 1,920 × 1,200; refresh rate: 59.95 Hz), which was controlled by a computer (Hewlett-Packard HP Compaq 8200 Elite CMT PC; processor: Intel@ Core™ i3-2100 CPU @ 3.10 GHz × 4; graphic card: Mesa Intel@ HD Graphics 2000) with an Ubuntu operating system (Ubuntu 22.04 LTS; GNOME version: 42.1). A special sound card (Creative Sound Blaster Audigy 2) was required for the multiple outputs. The computer program used to control the experiment was developed with Matlab (Mathworks Inc., Natick, MA, USA; Version R2022a) and the Psychophysics Toolbox 3 (Brainard, 1997; Kleiner et al., 2007; Pelli, 1997). The three loudspeaker sets were controlled separately using the PsychPortAudio functions. Figure 2 illustrates the set-up.

#### Stimuli

We used an auditory version of the Ternus display (Wang et al., 2014) with 200-ms sinewave tones (volume of 70 dB, measured at a distance of 5 cm). All tones were presented with a frequency of 800 Hz at one of three speaker locations. Between tones of a Ternus frame, a slight temporal offset (WFI) was used to ensure that the two sounds of a frame were not mixed together. We used a variable WFI of 10, 80, or 100 ms. In the experiment by Wang et al. (2014), a WFI of 10 ms was used for 50-ms tones. Instead of 50-ms tones we used 200-ms tones to ensure that the feature information in Experiments 2 and 3 will be sufficiently processed. As the WFI at which the sounds merge increases with the length of the sounds (e.g., Schubert & Wernick, 1969), we expected that we would need longer WFIs to avoid sound mixing.

#### Design and procedure

In Experiment 1, we conducted a 6 (ISI: 0, 25, 50, 100, 150, and 300 ms) × 3 (WFI: 10, 80, and 100 ms) within-subject design. All 18 conditions were repeated 20 times and the trials were completely counterbalanced per block and presented in a random order. Within each block, each condition combination was presented twice (36 trials per block). Overall, we presented ten blocks (one practice block and nine experimental blocks), leading to 360 trials per participant.

**Fig. 3:**
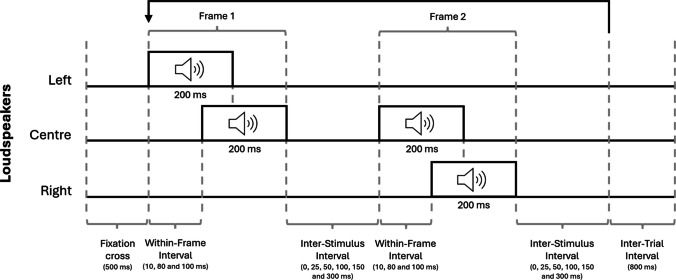
Trial sequence in all three experiments. First, a fixation cross was presented for 500 ms. This was followed by the first frame with a tone on the left side and in the center for 200 ms, separated by a within-frame interval (WFI; variable in Experiment 1, fixed at 80 ms in Experiments 2 and 3). After a variable inter-stimulus interval (ISI), the second frame was presented with a tone in the center and after the WFI on the right. The second frame was followed by the ISI, and the two stimulus frames and the ISI were presented in alternation until the participants responded. In Experiment 1 all tones were presented in the same frequency (800 Hz), in Experiments 2 and 3 a frequency-based feature bias was used (500 and 1,000 Hz). The next trial started after an inter-trial interval of 800 ms

After written instructions on the computer screen, the participants were shown a visual example of element and group motion using an ISI of zero and a long ISI (100 ms), respectively. After the visual example, the experimenter showed the participant an auditory example of element and group motion using an ISI of zero for element motion and a long ISI of 300 ms for group motion. Then the experiment started with a practice block followed by the experimental blocks. Figure 3 shows an example of the trial sequence. At the beginning of each trial, a fixation cross was presented at the monitor for 500 ms to ensure that participants were looking forward and the sound reaches the ears from a constant angle. This was followed by the auditory stream containing the first frame, the ISI, and the second frame. Within the first frame, a tone was presented on the left side. After a variable WFI (10, 80, or 100 ms), another tone was presented at the center. Within the second frame, a tone was presented at the center and, slightly delayed by the WFI, another tone was presented on the right side. Between the frames a variable ISI (0, 25, 50, 100, 150, or 300 ms) was presented. To ensure an ISI of 0 ms, tones 2 and 3 were merged to one tone of 400 ms. In contrast to Wang et al. (2014), we used a circular presentation of the stimuli, which means that the second frame was followed by the ISI and then by the first frame, and so on, until the participant responded. This was done to support a maximally stable percept, as is often done in the visual modality as well (e.g., Hein & Moore, 2012). As soon as the participant reacted, the cycle was interrupted and after an inter-trial interval of 800 ms, the next trial started. The participants were asked to indicate in each trial whether they perceived element (key J), group (key F), or no Ternus motion (key B). The answer option *no Ternus motion* was important because the WFI was manipulated in such a way that there were also conditions in which sound merging was supposed to take place within a frame. In such a case, the participants should only hear one sound per frame, which cannot be categorized as group or element motion. The experiment took 30 min.

#### Data analysis

The analysis for all experiments was done using RStudio (Version 2023.06.1 + 524, Posit Software, PBC, 2022). Within-subject analysis of variance (ANOVA) and pairwise two-sided post hoc *t*-tests were used to analyze the data in this and the other two experiments.^1^ Alpha was set at .05. When appropriate, reported *p*-values were Greenhouse–Geisser corrected (Greenhouse & Geisser, 1959) to correct for violations of the sphericity assumption in this and the following experiments. Shapiro–Wilk tests showed that group-motion as well as element-motion responses were normally distributed. Post hoc comparison *p*-values were adjusted according to Bonferroni. Standard deviations and standard errors were calculated for within-participants comparisons according to Cousineau (2005) and Morey (2008) using the summarySEwithin command from the Rmisc R package (Hope, 2013). In each experiment, the first block served as a practice block and was excluded from the analysis. In addition, all responses with key-presses other than the defined response keys (invalid key-presses), were excluded (0.04% of the data) in Experiment 1. Extreme reaction time (RT) outliers, i.e., all reactions that lasted longer than the mean reaction time + 5 SD across all participants were also excluded, as we assumed that participants were distracted from the task in these trials. We explicitly did not use a stricter cutoff criterion, as our participants were instructed to take as much time as they needed to respond while they were perceiving the cycling displays. In addition, participants tend to have longer RTs the more ambiguous the percept is, which were the data we were most interested in. That led to an exclusion of responses with a RT above 8.66 s (0.35% of the data) in Experiment 1. We performed ANOVAs on the percentage of perceived group-motion, element-motion, and no-motion responses. To calculate these percentages, we computed the proportion of group-/element-/no-motion responses for each condition out of all responses (group, element, and no motion).

### Results and discussion

**Fig. 4:**
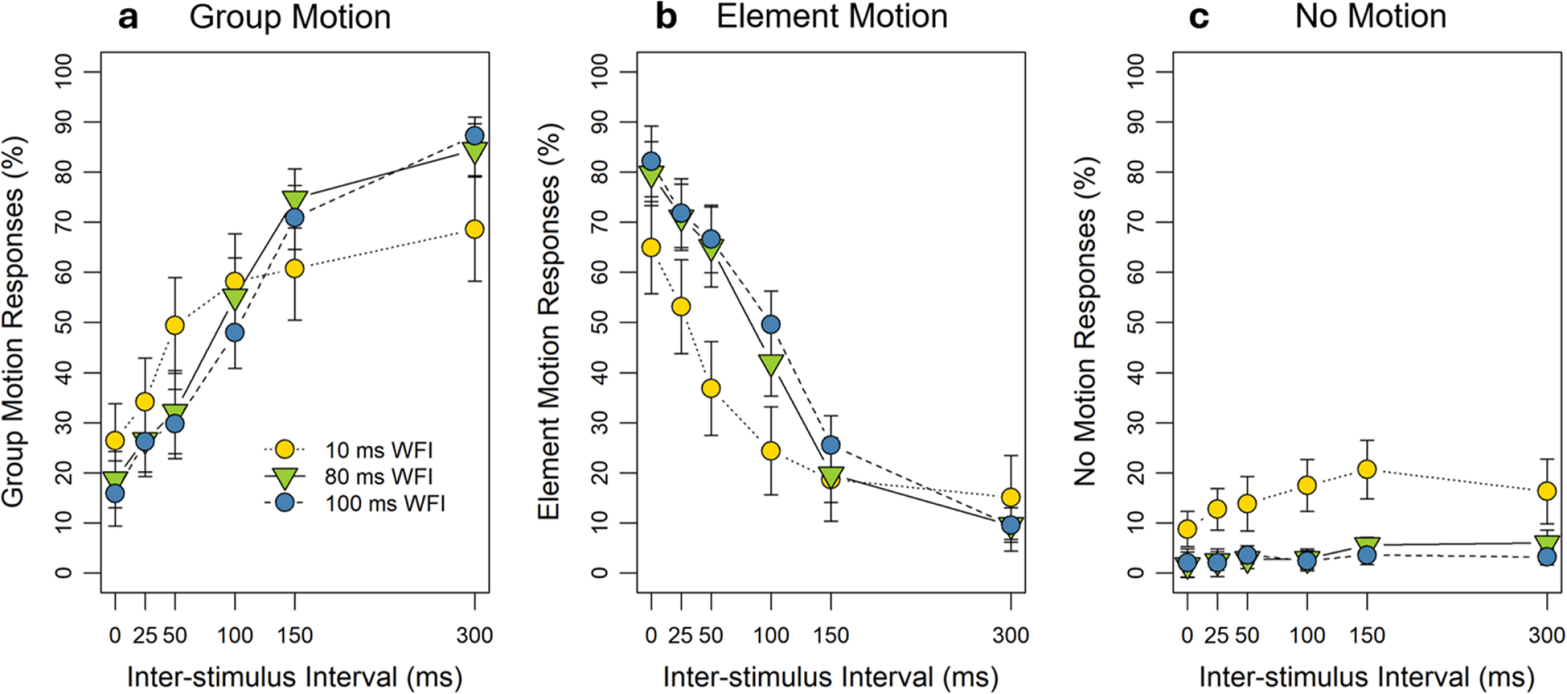
Perceived group, element, and no motion depending on the spatiotemporal factor inter-stimulus interval (ISI) and within-frame interval (WFI). Experiment 1: (**a**) Mean percent of perceived group motion as a function of ISI and WFI. (**b**) Mean percent of perceived element motion as a function of ISI and WFI. (**c**) Mean percent of perceived no motion as a function of ISI and WFI. The error bars represent within-subject standard errors (SEs) by Cousineau-Morey

Mean group-motion responses as a function of ISI and WFI are shown in Fig. 4a. We first conducted a two-factorial 6 (ISI: 0, 25, 50, 100, 150, and 300 ms) × 3 (WFI: 10, 80, and 100 ms) repeated-measures ANOVA on individual mean group-motion responses. The analyses showed a significant main effect of ISI, *F*(5, 65) = 31.50, *p* <.001, ${\eta }_{p}^{2}$ =.71,^2^ as group motion increased with increasing ISI. There was no significant main effect of the WFI, *F*(2, 26) = 0.06, *p* =.819, but the interaction between ISI and WFI was significant, *F*(10, 130) = 4.60, *p* =.004, ${\eta }_{p}^{2}$ =.26, as the influence of the ISI was weaker in the 10-ms WFI condition than in the other two conditions. Post hoc comparisons for the adjacent ISIs for each WFI separately showed that for the 10-ms WFI only the comparison between 25 and 50 ms was significant, *t*(13) = 3.70, *p* =.013, *d* = 0.99, *1˗β* = 0.93, while all other comparisons were not significant, *t*s ≤ 1.58, *p*s ≥.695. For the other two WFI conditions two comparisons were significant, between 50 and 100 ms (WFI 80: *t*(13) = 3.72, *p* =.013, *d* = 0.99, *1˗β* = 0.93; WFI 100: *t*(13) = 4.08, *p* =.006, *d* = 1.09, *1˗β* = 0.96), as well as between 100 and 150 ms (WFI 80: *t*(13) = 3.84, *p* =.010, *d* = 1.03, *1˗β* = 0.94; WFI 100: *t*(13) = 5.70, *p* <.001, *d* = 1.52, *1˗β* = 1.00). Thus, the interaction between WFI and ISI was based on group-motion responses increasing more rapidly with small ISIs, but then leveling quicker for the 10-ms WFI compared to the other two WFI conditions. All other comparisons for the adjacent ISIs were not significant (WFI 80: *t*s ≤ 2.06, *p*s ≥.299; WFI 100: *t*s ≤ 2.28, *p*s ≥.201).

Mean element-motion responses as a function of ISI and WFI are shown in Fig. 4b. We first conducted a two-factorial repeated-measures ANOVA on individual mean element-motion responses. The analyses showed a significant main effect of ISI, *F*(5, 65) = 33.23, *p* <.001, ${\eta }_{p}^{2}$ =.72, as element motion decreased with increasing ISI. There was no significant main effect of the WFI, *F*(2, 26) = 1.59, *p* =.229, but the interaction between ISI and WFI was significant, *F*(10, 130) = 4.59, *p* =.004, ${\eta }_{p}^{2}$ =.26, as the influence of the ISI was again weaker in the 10-ms WFI condition than in the other two conditions. Post hoc comparisons for adjacent ISIs for each WFI separately showed that for the 10-ms WFI only the comparison between 25 and 50 ms was significant, *t*(13) = 3.44, *p* =.022, *d* = 0.92, *1˗β* = 0.89. In addition, there was a trend for the comparison between 100 and 150 ms, *t*(13) = 2.95, *p* =.057, *d* = 0.79, *1˗β* = 0.78. All other comparisons were not significant, *t*s ≤ 2.30, *p*s ≥.195. For the other two WFI conditions, two comparisons were significant, between 50 and 100 ms (WFI 80: *t*(13) = 3.72, *p* =.013, *d* = 0.99, *1˗β* = 0.93; WFI 100: *t*(13) = 3.35, *p* =.026, *d* = 0.89, *1˗β* = 0.87), as well as between 100 and 150 ms (WFI 80: *t*(13) = 4.67, *p* =.002, *d* = 1.25, *1˗β* = 0.99; WFI 100: *t*(13) = 5.65, *p* <.001, *d* = 1.51, *1˗β* = 1.00). Thus, the interaction between WFI and ISI was based on element-motion responses decreasing more rapidly with small ISIs for the 10-ms WFI compared to the other WFI conditions, but then leveling with longer ISI for all WFIs. All other comparisons for the adjacent ISIs were not significant (WFI 80: *t*s ≤ 2.15, *p*s ≥.256; WFI 100: *t*s ≤ 2.28, *p*s ≥.201).

Overall, participants reported perceiving no motion in 7.07% of the trials. Mean no-motion responses are shown as a function of ISI and WFI in Fig. 4c. A two-factorial repeated-measures ANOVA for the factors ISI and WFI on individual mean no-motion responses showed that there was a significant main effect for the WFI, *F*(2, 26) = 5.27, *p* =.039, ${\eta }_{p}^{2}$ $=$.29. Post hoc comparisons for this factor revealed, however, no significant differences between the WFIs, *t*(13)s ≤ 2.34, *p*s ≥.107. Descriptively, participants reported more no motion in the 10-ms WFI condition than in the 80-ms and the 100-ms WFI conditions. In addition, the ANOVA showed that the no-motion percept did not change across the ISI, *F*(5, 65) = 1.57, *p* =.225. There was also no significant interaction between the WFI and the ISI, *F*(10, 130) = 0.68, *p* =.741.

The main results of this experiment, namely the spatiotemporal influence of the ISI on the auditory Ternus effect (increasing group-motion and decreasing element-motion responses with increasing ISI), replicated the results of Wang et al. (2014). In addition to that, our results showed that with a tone length of 200 ms the 10-ms WFI may have been too short to avoid sound mixing, because the 10-ms WFI condition was less affected by the spatiotemporal factor than the other WFI conditions for group- and element-motion responses and more no motion was perceived in the 10-ms WFI condition than in the other WFI conditions. It surprised us that the 10-ms WFI condition did not lead to many more no-motion responses, as under sound-mixing conditions only one object should be perceived in each frame, and thus both element and group-motion percepts should have been impossible. Several reasons might have contributed to this happening: First, participants might have expected to perceive either element or group motion, and therefore might have been reluctant to use the third answering option more often, convincing themselves that they must have perceived Ternus motion. Another explanation might be that the participants did not perceive element or group motion in the case of mixing, but still perceived motion, i.e., simple apparent motion between two elements, and therefore did not want to classify their percept as no motion, even though we instructed participants to use this option if they heard neither element nor group motion. The 80- and 100-ms WFIs on the other hand were sufficient to perceive Ternus motion in our experimental set-up, and this result is in line with research in this field (e.g., Litovsky et al., 1999; Schubert & Wernick, 1969). Therefore, we used an 80-ms WFI in the following experiments.

## Experiment 2: Frequency-based feature bias (mixed design)

In this experiment, we investigated the influence of feature information on auditory correspondence. In the visual modality, feature characteristics can have a strong influence on the correspondence process (e.g., Hein & Moore, 2012; Kramer & Yantis, 1997). To the best of our knowledge, there has been no research to date into whether a feature bias can influence correspondence in an ambiguous auditory apparent motion display. To investigate this question, we manipulated the frequency of the tones to create a feature-based bias (group, element, and no bias; see Fig. 1) in a similar way to Hein and Moore (2012) in the visual modality. We expected more group-motion percepts with a group bias and more element-motion percepts with an element bias. In addition to the feature bias, we tested six ISIs ranging from 0 to 300 ms, as in Experiment 1. As in the previous experiment, more group motion/less element motion should be perceived with longer ISIs and less group motion/more element motion with shorter ISIs.

### Materials and methods

#### Participants

As Experiment 1 showed that the ISI effect we found for the auditory Ternus was smaller than the one usually found for the visual modality, we decided to increase our sample size from 14 to 18 participants in Experiment 2 (and 3). A power analysis based on the visual feature-bias effect of the study by Hein and Moore (2012) found sample sizes between 2 and 5 were needed at least for a power of .8 and an alpha of .05. Participants who showed an inverse function of ISI across all feature-bias conditions (five participants) or pressed mostly only one key (one participant) were replaced. In addition, as we generally expected participants to be able to perceive motion in all conditions, people who indicated that they perceived no motion in more than 30% of the trials were replaced (two participants), as we assumed that these participants misunderstood the task. Based on these exclusion criteria, eight participants were replaced in Experiment 2. The final sample consisted of 18 participants (12 females; aged between 19 and 39 years, average age 22.44 years; 16 right-handers) in Experiment 2. Everything else was the same as in Experiment 1.

#### Equipment

The equipment was the same as in Experiment 1.

#### Stimuli

In Experiment 2 a feature bias was introduced using two tones with different frequencies for the Ternus display instead of the same frequencies for both tones as in Experiment 1. In particular, we used two sinewave tones with the frequencies of 500 and 1,000 Hz. In the no-bias condition, all tones were presented in the same frequency, in half of the trials at 500 Hz and in the other half of the trials at 1,000 Hz. For the group-bias condition, the tones from the three loudspeaker sets were compatible with the group-motion percept (Fig. 1c), thus the tone from the left loudspeaker in the first frame had the same frequency as the tone from the center loudspeaker in the second frame, and the tone from the center loudspeaker in the first frame had the same frequency as the tone from the right loudspeaker in the second frame, following the pattern A-B/A-B (A being one frequency and B the other frequency, the slash separates the tones into the two frames). For the element bias, the two tones were compatible with the element-motion percept (Fig. 1d), thus the tone from the left loudspeaker in the first frame and the tone from the right loudspeaker in the second frame had the same frequency, as well as the tone from the center loudspeakers in both frames, following the pattern A-B/B-A. In addition, we counterbalanced tone position, i.e., which frequency was presented at which loudspeaker in order to avoid potential systematic effects of the tone position: in half of the trials the low tone (500 Hz) was presented at the left loudspeaker set and in the other half of the trials the high tone (1,000 Hz). The WFI was fixed at 80 ms. Everything else was the same as in Experiment 1.

#### Design and procedure

In Experiment 2, we conducted a 6 (ISI: 0, 25, 50, 100, 150, and 300 ms) × 3 (Feature bias: group bias, element bias, and no bias) within-subject design, both factors were counterbalanced per block and presented in a random order. In each block, each factor combination was presented twice (36 trials per block). Overall, we presented 15 blocks (one practice block and 14 experimental blocks), leading to 540 trials per participant (30 trials per factor combination). Apart from that, the procedure was the same as in Experiment 1. The experiment took 45 min.

#### Data analysis

Data analysis was analogous to Experiment 1, eliminating invalid key-presses and using the same RT cutoff of + 5 SD. Based on responses with invalid key-presses, 0.07% of the data was excluded. In addition, responses with a RT above 11.49 s (0.25% of the data) were excluded in Experiment 2. The position was a methodical factor and thus was not considered in the data analysis. Shapiro–Wilk tests showed that feature bias, our main factor of interest, was normally distributed for group-motion as well as element-motion responses.

### Results and discussion

**Fig. 5:**
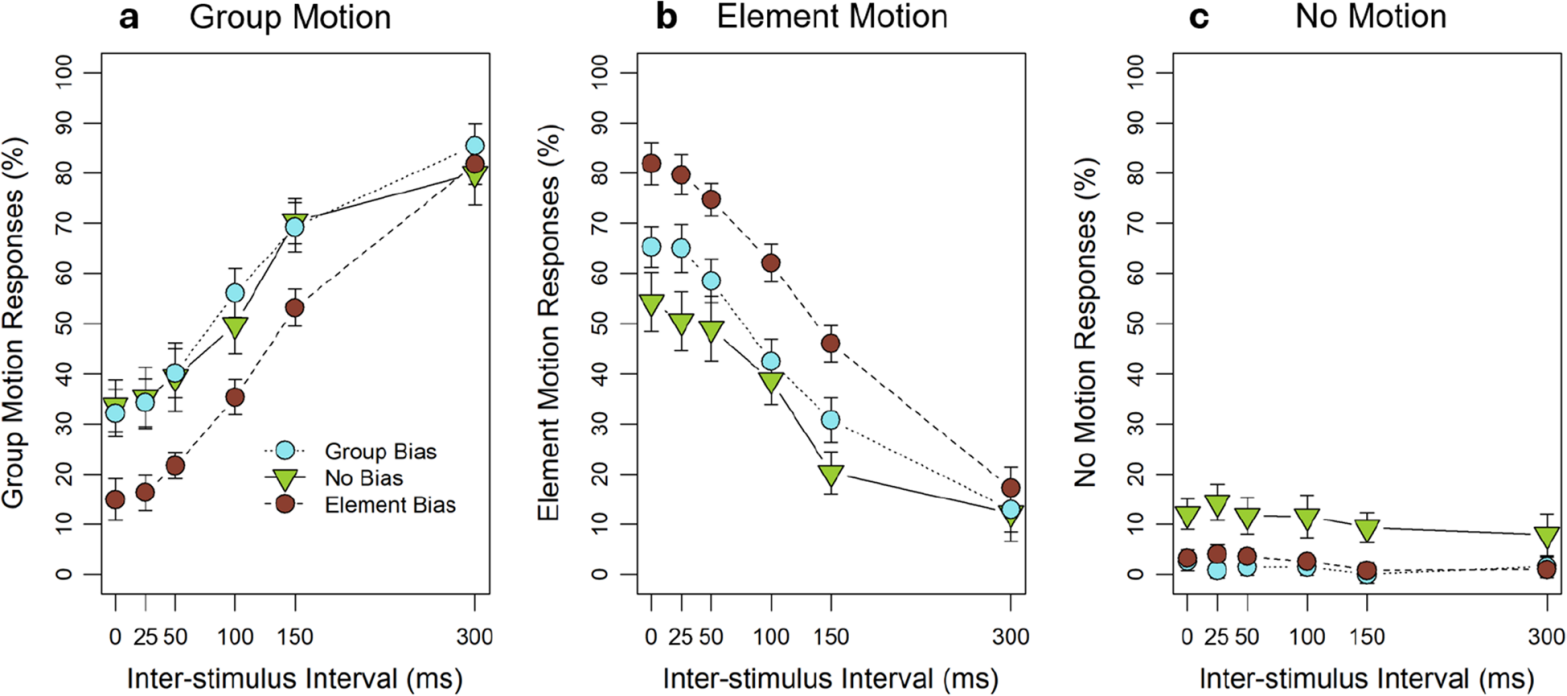
Perceived group, element, and no motion depending on the spatiotemporal and frequency-based feature factors (mixed design). Experiment 2: (**a**) Mean percent of perceived group motion as a function of inter-stimulus interval (ISI) and Feature bias (group, element and no bias). (**b**) Mean percent of perceived element motion as a function of ISI and Feature bias. (**c**) Mean percent of perceived no motion as a function of ISI and Feature bias. The error bars represent within-subject standard errors (SEs) by Cousineau-Morey

Mean group-motion responses are shown as a function of ISI and Feature bias in Fig. 5a. We first conducted a two-factorial 6 (ISI: 0, 25, 50, 100, 150, and 300 ms) × 3 (Feature bias: group bias, element bias, and no bias) repeated-measures ANOVA on individual mean group-motion responses. Consistent with our first experiment, the analyses showed a significant main effect of ISI, *F*(5, 85) = 54.21, *p* <.001, ${\eta }_{p}^{2}$ =.76, as group motion increased with increasing ISI. Post hoc comparisons for adjacent ISIs revealed that this effect was based on significant differences between the last four ISI levels, *t*s(17) ≥ 5.02, *p*s <.001, *d*s ≥ 1.18, *1˗β* = 1.00. There were no differences between the first three ISI levels, *t*s(17) ≤ 2.39, *p*s ≥.145, *d*s ≤ 0.56. In addition to the ISI effect, we found a significant main effect of the Feature bias,* F*(2, 34) = 5.18, *p* =.024, ${\eta }_{p}^{2}$ =.23. Most importantly, post hoc comparisons for this factor revealed a significant difference between the group- and element-bias condition, *t*(17) = 4.21, *p* =.002, *d* = 0.99,* 1˗β* = 0.98, as more group-motion percepts were reported for the group-bias compared to the element-bias condition. In addition, the no-bias and element-bias conditions differed significantly from each other, *t*(17) = 2.82, *p* =.035, *d* = 0.67, *1˗β* = 0.76, as more group-motion percepts were reported in the no-bias compared to the element-bias condition. The group-bias and the no-bias conditions, however, did not differ significantly from each other, *t*(17) = 0.22, *p* = 1. The ANOVA also revealed a trend for the interaction between Feature bias and ISI,* F*(10, 170) = 2.24, *p* =.071, ${\eta }_{p}^{2}$ =.12, as the bias effect decreased with increasing ISI, disappearing for the longest ISI condition.

Mean element-motion responses are shown as a function of ISI and Feature bias in Fig. 5b. We first conducted a two-factorial repeated-measures ANOVA on individual mean element-motion responses. Consistent with our first experiment, the analyses showed a significant main effect of ISI, *F*(5, 85) = 49.62, *p* <.001, ${\eta }_{p}^{2}$ =.74, as element motion decreased with increasing ISI. Post hoc comparisons for adjacent ISIs revealed that this effect was based on significant differences between the last four ISI levels, *t*s(17) ≥ 5.13, *p*s <.001, *d*s ≥ 1.21, *1˗β* = 1.00. There were no differences between the first three ISI levels, *t*s(17) ≤ 2.14, *p*s ≥.235, *d*s ≤ 0.50. In addition to the ISI effect, we found a significant main effect of the Feature bias,* F*(2, 34) = 10.32, *p* <.001, ${\eta }_{p}^{2}$ =.38. Most importantly, post hoc comparisons for this factor revealed a significant difference between the group- and the element-bias condition, *t*(17) = 3.90, *p* =.003, *d* = 0.92,* 1˗β* = 0.96, as more element-motion percepts were reported for the element-bias compared to the group-bias condition. In addition, the no-bias and element-bias conditions differed significantly from each other, *t*(17) = 4.54, *p* <.001, *d* = 1.07, *1˗β* = 0.99, as more element-motion percepts were found in the element-bias compared to the no-bias condition. The group-bias and the no-bias conditions, however, did not differ significantly from each other, *t*(17) = 1.35, *p* = 0.588. The ANOVA also revealed a significant interaction between Feature bias and ISI,* F*(10, 170) = 2.78, *p* =.032, ${\eta }_{p}^{2}$ =.14, as the bias effect decreased with increasing ISI, disappearing for the longest ISI condition.

We repeated the two-factorial repeated-measures ANOVA (ISI × Feature bias) on percent no-motion responses. Overall, participants reported to perceive no motion in 4.98% of the trials. We found a significant main effect of Feature bias, *F*(2, 34) = 5.41, *p* =.031, ${\eta }_{p}^{2}$ =.24, as the no-bias condition led to more no-motion percepts than the group- and element-bias conditions (see Fig. 5c). Beside this feature-bias effect, the perceived no motion did not change across the ISIs and the interaction with the feature bias was also not significant (*F*s ≤ 1.13, *p*s ≥.325).

The results for the group- and element-motion responses replicated the strong influence of the ISI on the perception of the Ternus display. This result is consistent with Experiment 1 and in line with the literature (Wang et al., 2014). Most importantly, we obtained a strong effect of the feature bias, as the group bias led to more group-/less element-motion percepts than the element bias. Thus, a frequency-based bias influenced correspondence in the auditory Ternus, suggesting that features play a role in solving correspondence in the auditory modality, as has been shown in the visual modality (e.g., Hein & Moore, 2012; Kramer & Yantis, 1997). Interestingly, the group bias in our experiment was not independent from the ISI as Hein and Moore (2012) found for the visual modality. This suggests that the influence of the feature bias did not overwrite the influence of the ISI in the auditory Ternus; instead we found a strong influence of the ISI in all three feature-bias conditions. In addition, the no-bias condition seemed to be somehow special, as no motion was more often perceived in the no-bias condition compared to the other two bias conditions. One reason for that could be that focusing on the feature information, participants might have grouped the stimuli within an auditory frame as they both had the same feature in the no-bias condition, similar to what has been proposed for the visual modality (Kramer & Rudd, 1999; Kramer & Yantis, 1997). This binding would result in one auditory object per frame, which cannot be perceived as either element or group motion and which therefore might have let participants choose the third response option, as they might have perceived simple apparent motion. Overall, our results strengthen the assumption that the correspondence mechanism works similarly in the auditory and visual modalities.

## Experiment 3: Frequency-based feature bias (blocked design)

In Experiment 2 we replicated the typical effect of the ISI for the auditory Ternus display, and we showed for the first time that a feature bias can influence correspondence in an auditory apparent motion display. In this experiment we wanted to replicate the bias effect and see whether it is stable across different presentation modes and thus different contexts. We used a blocked design to present the different feature biases separately in different blocks. In addition, using another presentation mode allowed us to investigate the feature bias further and the special pattern of the no-bias condition. We reasoned that in a blocked design the task might be easier, as the participants can concentrate on one bias condition, which might lead to a stronger feature bias and a no-bias condition that is more similar to the 80-ms WFI condition in Experiment 1. Apart from the blocked design the experiment was identical to Experiment 2. As in the previous experiment, we varied the frequency-based feature bias (group, element and no bias) and tested six ISIs ranging from 0 to 300 ms. As in the previous experiment we expected more group-motion percepts with a group bias and with a longer ISI and more element-motion percepts with an element bias and with a shorter ISI.

### Materials and methods

#### Participants

The sample size was the same as in Experiment 2. Participants who showed an inversive function of the ISI (five participants) or pressed mostly only one key (one participant) were replaced. No participant perceived no motion in more than 30% of the trials. Based on these exclusion criteria, six participants were replaced in Experiment 3. The final sample consisted of 18 participants (15 females; aged between 19 and 36 years, average age 24 years; 16 right-handers). Everything else was the same as in Experiment 2.

#### Equipment and stimuli

The equipment and stimuli were the same as in Experiment 2.

#### Design and procedure

As in the previous experiment, in Experiment 3 we conducted a 6 (ISI: 0, 25, 50, 100, 150, and 300 ms) × 3 (Feature bias: group bias, element bias, and no bias) within-subject design. In contrast to Experiment 2, in which the feature bias was randomly intermixed, the feature bias was presented in a blocked design. Each feature-bias condition was counterbalanced with the ISI and presented in a random order in each block. Overall, we presented 13 blocks (one practice block with 24 trials and 12 experimental blocks with 36 trials), leading to 432 trials per participant (24 trials per factor combination). One bias condition was always presented for four consecutive experimental blocks. The bias used in the practice block corresponded to the bias of the first four experimental blocks and was thus balanced across participants. The order of the three biases was also balanced across participants. Apart from that the procedure was the same as in Experiment 2.

#### Data analysis

Based on responses with invalid key-presses, 0.41% of the data was excluded. In addition, responses with a RT above 9.95 s (0.39% of the data) were excluded using the same RT cutoff of + 5 SD as in the other two experiments. Shapiro–Wilk tests showed no violation of normality for our main factor of interest (feature bias). Apart from that the data analysis was the same as in Experiments 1 and 2.

### Results and discussion

**Fig. 6:**
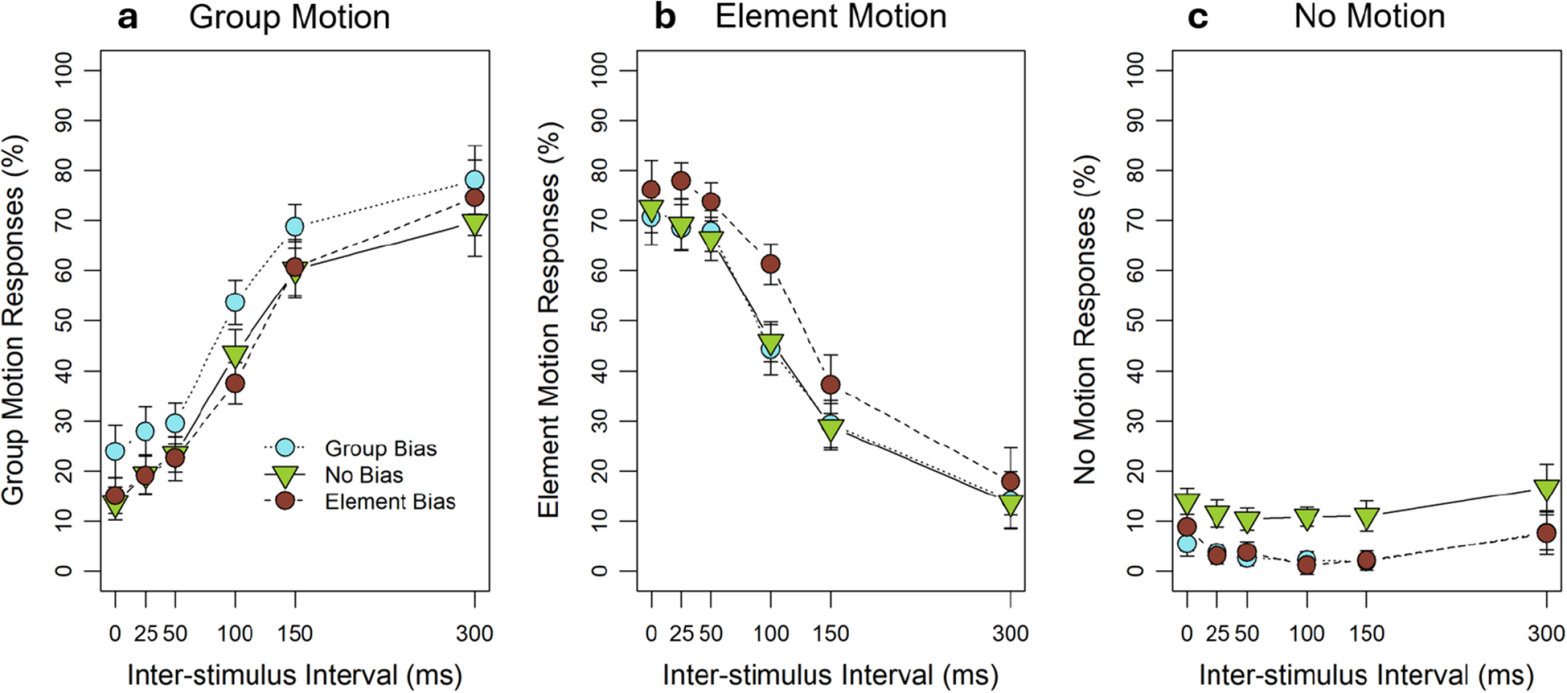
Perceived group, element, and no motion depending on the spatiotemporal and frequency-based feature factors (blocked design). Experiment 3: (**a**) Mean percent of perceived group motion as a function of inter-stimulus interval (ISI) and Feature bias (group, element, and no bias). (**b**) Mean percent of perceived element-motion as a function of ISI and Feature bias. (**c**) Mean percent of perceived no motion as a function of ISI and Feature bias. The error bars represent within-subject standard errors (SEs) by Cousineau-Morey

Mean group-motion responses are shown as a function of ISI and Feature bias in Fig. 6a. We first conducted a two-factorial 6 (ISI: 0, 25, 50, 100, 150 and 300 ms) × 3 (Feature bias: group bias, element bias and no bias) repeated-measures ANOVA on individual mean group-motion responses. Consistent with our previous experiments, the ANOVA showed again that with increasing ISI the proportion of group-motion responses increased, *F*(5, 85) = 29.10, *p* <.001, ${\eta }_{p}^{2}$ =.63. Post hoc comparisons for adjacent ISIs revealed that the following ISI levels were significantly different from each other: 0 and 25 ms, *t*(17) = 3.07, *p* =.035, *d* = 0.72, *1˗β* = 0.82, 50 and 100 ms, *t*(17) = 3.91, *p* =.005, *d* = 0.92, *1˗β* = 0.96, and 100 and 150 ms, *t*(17)$=$ 5.02, *p* <.001, *d* = 1.18, *1˗β* = 1.00. The other two comparisons, 25 and 50 ms as well as 150 and 300 ms, were not significant, *t*(17)s ≤ 2.05, *p*s ≥.282. In addition, we found a main effect of Feature bias, *F*(2, 34) = 6.65, *p* =.004, ${\eta }_{p}^{2}$ =.28. Post hoc comparisons revealed that group-motion responses were significantly higher for the group bias compared to the no bias, *t*(17) = 2.93, *p* =.028, *d* = 0.69, *1˗β* = 0.79, and compared to the element bias, *t*(17) = 3.79, *p* =.004, *d* = 0.89, *1˗β* = 0.94. The no-bias and element-bias conditions did not differ significantly from each other, *t*(17) = 0.01, *p* = 1. The interaction between ISI and Feature bias was not significant, *F*(10, 170) = 0.57, *p* =.737.

Mean element-motion responses are shown as a function of ISI and Feature bias in Fig. 6b. We first conducted a two-factorial repeated-measures ANOVA on individual mean element-motion responses. Consistent with our previous experiments, the ANOVA showed again that with increasing ISI the proportion of element-motion responses decreased, *F*(5, 85) = 31.22, *p* <.001, ${\eta }_{p}^{2}$ =.65. Post hoc comparisons for the adjacent ISIs revealed that the following ISI levels were significantly different from each other: 50 and 100 ms, *t*(17) = 3.59, *p* =.011, *d* = 0.85, *1˗β* = 0.92, 100 and 150 ms, *t*(17) = 4.97, *p* <.001, *d* = 1.17, *1 – β* = 1.00, and 150 and 300 ms, *t*(17)$=$ 4.07, *p* =.004, *d* = 0.96, *1˗β* = 0.97. The other two comparisons, 0 and 25 ms as well as 25 and 50 ms, were not significant, *t*(17)s ≤ 1.50, *p*s ≥.761. In addition, we found a main effect of Feature bias, *F*(2, 34) = 5.99, *p* =.006, ${\eta }_{p}^{2}$ =.26. Post hoc comparisons revealed that element-motion responses were significantly higher for the element bias compared to the no bias, *t*(17) = 2.95, *p* =.027, *d* = 0.70, *1 – β* = 0.80, and compared to the group bias, *t*(17) = 3.41, *p* =.010, *d* = 0.80, *1 – β* = 0.89. The no-bias and group-bias conditions did not differ significantly from each other, *t*(17) = 0.08, *p* = 1. The interaction between ISI and Feature bias was not significant, *F*(10, 170) = 0.84, *p* =.529.

We performed another repeated-measures two-factorial ANOVA (ISI × Feature bias) on individual mean no-motion responses. Overall, participants reported to perceive no motion in 6.88% of the trials. We found no main effect for the ISI, *F*(5, 85) = 1.56, *p* =.227, but a significant main effect of the Feature bias, *F*(2, 34) = 7.58, *p* =.005, ${\eta }_{p}^{2}$ =.31. Post hoc comparisons showed a significant difference between the no-bias and the group-bias condition, *t*(17) = 3.15, *p* =.017, *d* = 0.74, *1˗β* = 0.84, as well as between the no-bias and the element-bias condition, *t*(17) = 2.81, *p* =.036, *d* = 0.66, *1˗β* = 0.75, as more no-motion responses were given in the no-bias condition compared to the group- and element-bias conditions (see Fig. 6c). The element- and group-bias conditions did not differ significantly from each other, *t*(17) = 0.31,* p* = 1. The ANOVA showed no interaction between ISI and Feature bias, *F*s(10, 170) = 0.29, *p* =.813.

**Fig. 7:**
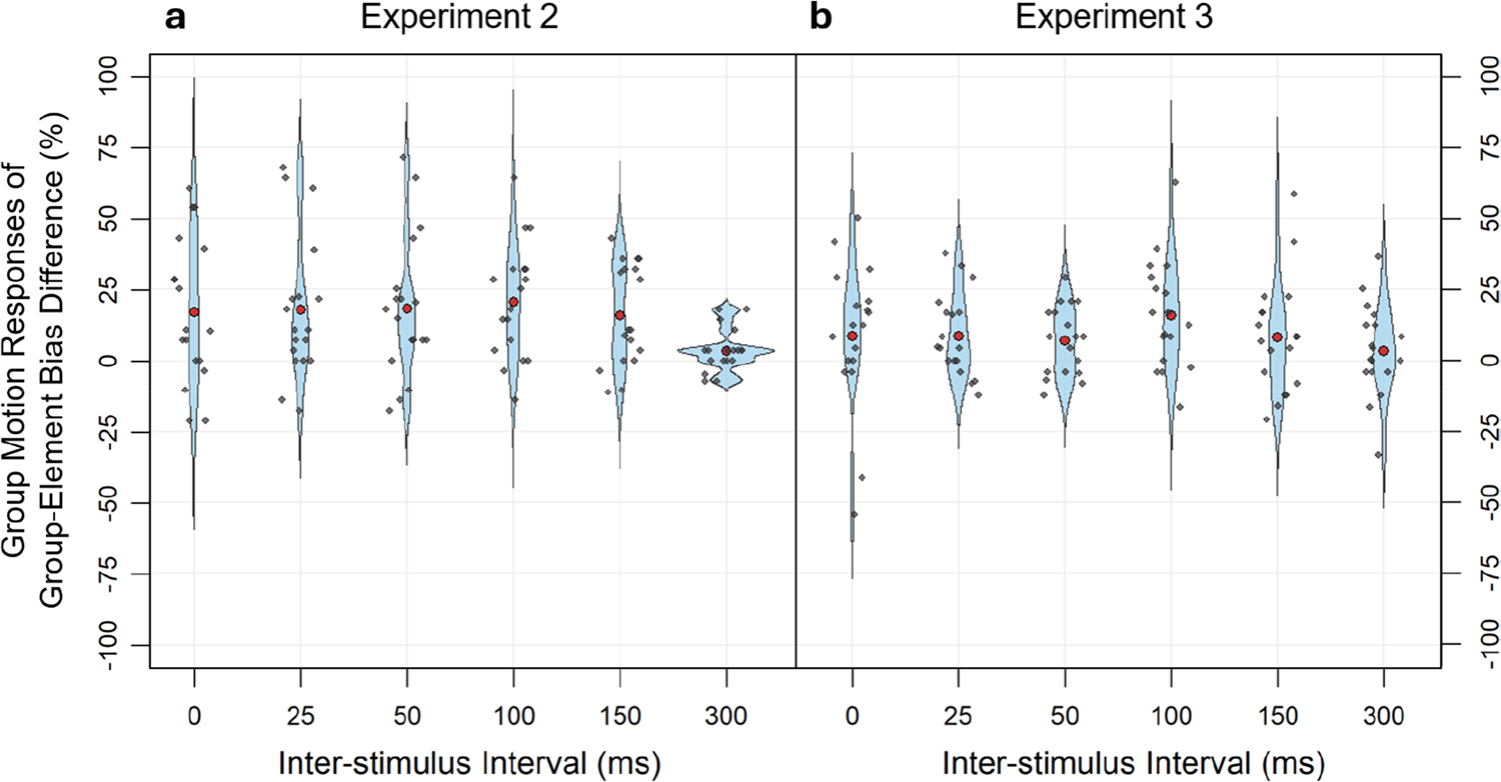
Violin plots of the bias effect for group-motion responses. The small dots represent individual differences in perceived group motion between the group and element bias and the big red dots represent the mean difference averaged over participants. (**a**) In Experiment 2, the factors inter-stimulus interval (ISI) and Feature bias were presented in a mixed design. (**b**) In Experiment 3, the factors ISI and Feature bias were presented in a blocked design

In line with the previous experiments and the literature (Wang et al., 2014), we replicated the ISI effect. Importantly, the feature bias showed again a significant influence on motion percepts in the blocked design, as the group bias led to more group-motion/less element-motion percepts than the element bias. Additionally, this feature effect was even more stable across the ISI than in Experiment 2 (see Fig. 7). Concentrating on one bias condition within a block apparently did not make the task easier for the participants and had no effect on the no-bias condition. The replication of the feature effect underscores the strong influence of the feature bias on auditory motion perception: It persists in this blocked design even though participants might have focused more on the variable ISI within a block than on feature bias, which remained constant within a block, showing that the auditory feature-bias effect is robust across different presentation contexts. Finally, as in the previous experiment, more no-motion responses were perceived in the no-bias condition compared to the other two bias conditions. In sum, Experiment 3 replicated the influence of the feature bias from Experiment 2, strengthening the conclusion that a frequency-based bias can influence correspondence in the auditory modality.

## General discussion

Perceptual correspondence is the process that connects different instances of an object across space and time and thus creates stable object representations. Our goal for this series of experiments was to investigate whether the visual and auditory correspondence solutions are influenced by similar factors. To this end, we used an auditory Ternus display, in which two different apparent motion percepts, group or element motion, can be heard (Wang et al., 2014), depending on how correspondence has been established. Studies have shown that spatiotemporal information can influence correspondence in the Ternus display in vision (e.g., Pantle & Picciano, 1976; Petersik & Pantle, 1979), audition (Wang et al., 2014), and touch (Harrar & Harris, 2007). In the visual modality, feature information can additionally influence correspondence (e.g., Hein & Moore, 2012; Kramer & Yantis, 1997), but whether feature information is also important for solving the correspondence process in other modalities has not been investigated. To reduce this knowledge gap, we introduced a frequency-based feature bias in an auditory Ternus display similar to what has been done in the visual modality (e.g., Hein & Moore, 2012; Kramer & Yantis, 1997). If feature information can influence auditory correspondence, we expected to find more group motion in the group-bias condition, as the percept is compatible with group motion, as well as more element motion in the element-bias condition, as the percept is compatible with the element motion. We also expected to replicate the spatiotemporal effect, i.e., more group-motion and less element-motion percepts with longer ISIs (Wang et al., 2014). In Experiments 1–3 we confirmed the effect of spatiotemporal information on the auditory Ternus. In Experiments 2 and 3, we showed that feature information can influence auditory correspondence in line with our hypothesis. This feature bias effect on auditory motion perception was independent of the presentation mode (mixed or blocked) and thus it can be assumed to be a stable factor of influence.

Our experiments showed that both spatiotemporal and feature information influence correspondence in the auditory modality, as has been shown in the visual modality. It is possible that the mechanisms to solve correspondence have developed analogously within the two modalities and thus the apparent motion percepts were influenced by similar factors. In contrast to such separate mechanisms in each modality, similar factors of influence might also be interpreted as evidence for the existence of a multimodal processing mechanism. Such a mechanism might be able to integrate information from different modalities. There is lot of evidence in support of multimodal processing of visual and auditory stimuli, establishing a uniform percept across different sensory modalities (e.g., Burr & Morrone, 2011; Calvert, 2001; Ernst & Bülthoff, 2004; Giard & Peronnet, 1999; King & Calvert, 2001; Macaluso & Driver, 2005; Meredith & Stein, 1983; Stein & Stanford, 2008; Wallace et al., 1998). For apparent motion in particular, studies have shown that information from different modalities interacts, influencing the apparent motion percept (auditory-visual: Sanabria et al., 2005a; Shi et al., 2010; auditory-tactile: Chen et al., 2011; tactile-visual: Harrar & Harris, 2007; auditory-visual-tactile: Sanabria et al., 2005b). For example, Sanabria et al. (2005b) investigated whether the perception of simple auditory apparent motion (a tone from the left followed by a tone from the right or vice versa) can be affected by visual and/or tactile apparent motion distractors presented either in a congruent or incongruent motion direction. They showed that the detection of the auditory apparent motion direction was better in congruent than incongruent conditions independent of the distractor modality. These results suggest that irrelevant information from different modalities presented simultaneously can influence the percept of apparent motion of another target modality.

The question remains, however, how a multimodal correspondence mechanism might work. In the visual modality different theories have been proposed to explain how correspondence is established (Hein, 2017; Petersik & Rice, 2006). Motion-based theories assume that spatiotemporal information is dominant for solving correspondence and that correspondence could work through a very simple low-level mechanism based on motion detectors or spatiotemporal filters (e.g., Adelson & Bergen, 1985; van Santen & Sperling, 1985). Object-based theories, on the other hand, propose that although spatiotemporal information can be used for establishing correspondence, the feature information of the objects, such as their color or shape, is at least as important as spatiotemporal information. Following these theories, correspondence is resolved based on the (perceived) similarity of the objects (e.g., He & Ooi, 1999; Hein & Cavanagh, 2012; Hein & Moore, 2012, 2014; Kramer & Rudd, 1999; Ramachandran et al., 1998; Stepper et al., 2020a). Even features that are not directly part of the object, as for example prior knowledge about object properties, can affect correspondence (Stepper et al., 2020b). These findings support the need for object-based theories, as motion-based theories cannot explain them. It is likely, however, that both motion-based and object-based mechanisms play a role in visual correspondence. A question that remains to be answered is whether both types of correspondence also influence auditory correspondence. Our finding that auditory correspondence is not only influenced by spatiotemporal but also by feature information supports the assumption that both mechanisms might play a role in auditory correspondence as well. But studies that directly investigate the possible impact of higher-level object information, as for example prior knowledge about the object, remain to be done in the auditory modality.

The feature-bias effect that we found in our experiments was more dependent on the ISI and less strong than the effect usually found in the visual modality. This attenuation of the feature bias in the auditory modality might be due to differences in the strength of the feature bias between the modalities. Alternatively, this difference could be caused by the primary sense of humans being visual, and humans typically relying on visual stimuli in everyday life. As a result, our hearing may not be as well trained as our vision. It would therefore be interesting to investigate auditory correspondence in visually impaired people and musicians, as compared to normally sighted people the auditory sense should play a more important role in visually impaired/blind people (e.g., Kolarik et al., 2017; Voss et al., 2008) and can be enhanced through musical training (e.g., Kraus & Chandrasekaran, 2010). Thus, it might be possible that people with a visual impairment and musicians have an auditory apparent motion percept that is more like the visual one in sighted people and people without extensive music exposure in terms of the strength and independence of the ISI.

In conclusion, our study showed that spatiotemporal and feature-based factors influence the way the brain establishes correspondence between acoustic signals and thus perceives auditory objects as moving over time. The finding that both factors influence auditory correspondence and in particular the influence of a feature bias in the auditory and in the visual modality suggests that both modalities might use similar or even the same overarching mechanisms to connect objects across space and time. Future studies will have to further investigate the nature of these mechanisms and whether they are multimodal, i.e., integrating information from different modalities. In sum, the results of the present experiments expand our knowledge by showing that feature information is used in the auditory modality to generate a continuous representation of moving auditory objects.

## Data Availability

The data are available at zenodo.org (10.5281/zenodo.17083847). None of the experiments was preregistered.

## Footnotes

1 Based on the suggestion of a reviewer, we also ran multinomial logistic regression analysis for all three experiments to directly compare all three response options with each other. These analyses confirmed the results of the ANOVAs.

2 We also analyzed our data without replacing the participants who showed an inversive function of ISI and still found a strong effect of the ISI, *F*(5, 65) = 10.70, *p* =.001, ${\eta }_{p}^{2}$=.45.
